# A phase 1 evaluation of the safety and tolerability of niraparib in combination with everolimus in advanced ovarian and breast cancers

**DOI:** 10.1002/cam4.6475

**Published:** 2023-08-30

**Authors:** David Starks, Luis Rojas‐Espaillat, Tobias Meissner, Rachel Elsey, Bing Xu, Maria Koenen, Shelley Feng, Annika VanOosbree, John Slunecka, John Lee, Casey B. Williams

**Affiliations:** ^1^ Avera Cancer Institute Sioux Falls South Dakota USA; ^2^ University of South Dakota Sanford School of Medicine Sioux Falls South Dakota USA

**Keywords:** everolimus, niraparib, ovarian cancer, women's cancer

## Abstract

**Objectives:**

Phase 1 trial to determine the safety and tolerability of everolimus and niraparib in patients with advanced ovarian and breast malignancies.

**Results:**

Fourteen heavily pretreated patients were enrolled (12 high‐grade serous ovarian cancer, 1 clear cell ovarian cancer, and 1 triple negative breast cancer). All patients were PARP naïve and received comprehensive genomic profiling prior to enrollment. Two DLTs were experienced in cohort 2 (niraparib 200 mg daily and everolimus 5 mg 3 days per week) with one patient experiencing prolonged thrombocytopenia and the other experiencing severe hypertension. Four additional patients were enrolled after dose de‐escalation with one patient again experiencing severe hypertension leading to conclusion of the study. The most frequent grade 3 or greater adverse events were thrombocytopenia, hypertension, anemia, fatigue, neutropenia, and elevated alkaline phosphatase. Two patients had a PR and five patients had SD. ORR was 18% and the CBR was 45% in 11 evaluable patients. Median PFS was 6 months, and median OS is approximately 18 months with three patients still alive at the data cutoff.

**Conclusions:**

The combination of everolimus and niraparib demonstrated significant toxicity at lower doses and is not feasible due to rapid onset and severe hypertension. This limitation possibly blunted the efficacy of the combination as PFS was modest, but OS was surprisingly robust due to three patients with ovarian cancer remaining alive with platinum refractory disease. Further investigation of multiagent blockade of the PI3K pathway combined with PARP is warranted.

## 1 INTRODUCTION

Poly (adenosine diphosphate [ADP]‐ribose) polymerases (PARP) are enzymes that play a significant role in DNA repair, promoting cell survival by activating the base excision repair and single‐strand break repair pathways. Inhibition of PARP can prevent proper repair and lead to DNA double‐strand breaks that cannot be properly repaired, a situation found in tumors that have defects in the homologous recombination pathway (HRD), or mutations in *BRCA1* or *BRCA2*. Failure to properly repair the DNA damage leads to tumor cell death. Germline mutations in *BRCA1* and *BRCA2* are found in roughly 17% of women with epithelial ovarian cancer (EOCs) and 10%–20% of triple negative breast cancers (TNBCs), while somatic mutations are found in approximately 7% of ovarian tumors and 3%–5% of TNBCs.^1^, ^2^, ^3^, ^4^, ^5^, ^6^ Roughly 40%–50% of EOCs have HR deficiencies, while HRD is a frequent occurrence in TNBCs.^5^, ^6^, ^7^, ^8^, ^9^ PARP inhibition of tumors with HRD has led to a dramatic change in the management of EOCs with statistically and clinically significant improvements in outcomes.^9^, ^10^, ^11^, ^12^ Indeed, PARP maintenance after completion of upfront platinum‐based chemotherapy has become the standard of care for EOC patients with germline or somatic BRCA mutations or HR deficient tumors.

The PI3K/AKT/mTOR (mammalian Target of Rapamycin) pathway is responsible for several important cellular processes crucial to maintaining homeostasis in the cell. This pathway, however, is a frequent site of mutations that can lead to cancer development and growth. The PI3K/AKT/mTOR pathway is altered in many human cancers, including breast and ovarian cancers, and has been extensively studied as a target for anticancer therapies for many years.^13^, ^14^, ^15^, ^16^, ^17^ The mTOR complex is a serine/threonine kinase that plays a large role in the protein synthesis required for cell growth. Inhibition of this complex may prevent abnormal cell proliferation and angiogenesis that could sustain a tumor. Durable blockade of this pathway with single agent drugs has been found to be limited due to short‐term efficacy in many patients, as well as increased toxicity.^18^, ^19^, ^20^, ^21^ Combinations with other chemotherapeutic agents and different dosing schedules are being investigated to overcome the limitations of short‐term efficacy and intolerable toxicity. Several studies have demonstrated, both in vitro and in vivo, the role PI3K and mTOR play in double strand DNA repair, and it has been shown that PI3K‐mTOR inhibition can sensitize *BRCA1* mutated TNBC to PARP inhibition. This suggests an overlap between PARP‐mediated repair and the PI3K pathway.^7^, ^22^, ^23^, ^24^, ^25^

Everolimus is a first‐generation mTOR inhibitor that binds the intercellular receptor FKB12, which then binds and preferentially inactivates the mTOR protein kinase complex 1 (mTORC1) slowing tumor growth, limiting metastatic and local spread, and inhibiting DNA damage repair. However, this pathway disruption does activate feedback mechanisms that can limit its effectiveness and cause side effects. In a large meta‐analysis, the most common side effects of everolimus therapy were found to be stomatitis, leukopenia, anorexia, anemia, and fatigue.^26^ Most of these adverse effects were grade 1 or 2 as classified by Common Toxicity Criteria for Adverse Events (CTCAE) scale of the National Cancer Institute, indicating that the drug is overall reasonably tolerated as a single agent.^27^

Niraparib is an orally available poly (adenosine diphosphate [ADP]–ribose) polymerase (PARP) 1/2 inhibitor that is currently approved in the United States as first‐line maintenance therapy for patients with advanced or recurrent platinum‐sensitive ovarian cancer regardless of BRCA status who have had a complete or partial response to platinum‐based chemotherapy. Niraparib is also approved for second‐line maintenance in patients with germline BRCA mutations after treatment for platinum‐sensitive recurrent disease. Niraparib was previously indicated for patients with homologous recombination deficiency‐associated (HRD‐associated) epithelial ovarian cancer who had previously undergone three or more chemotherapy cycles, but this indication was withdrawn in 2022 after results from Quadra, as well as Ariel 4 and SOLO 3 studies, demonstrated a decreased OS in patients receiving PARP treatment as opposed to chemotherapy.^10^, ^11^, ^28^, ^29^ The safety profile of niraparib has been well documented in several large Phase 3 trials.^28^ Dosing is 300 mg orally on a daily basis with the ability to dose reduce to 200 mg daily for patients weighing less than 170 pounds, platelet count <150,000/μL or to improve patient tolerability. The most common adverse reactions were thrombocytopenia (66%), anemia (64%), nausea (57%), fatigue (51%), neutropenia (42%), and constipation (40%). Myelodysplastic syndrome/acute myeloid leukemia was reported in 1.2% of treated patients.

In generating lethal double‐strand DNA breaks, PARP inhibition has proven to be a very effective target in the management of multiple malignancies. The PI3K pathway appears to contribute to the repair of double‐strand DNA breaks by maintaining a homologous recombination steady state.^30^ Blocking PI3K function has been shown to lead to an HRD state. Preclinical data from our group and others have demonstrated the combination of a PI3K/mTOR inhibitor with a PARP inhibitor is synergistic in multiple cancer cell lines.^15^, ^30^, ^31^, ^32^ The combination of a PARP inhibitor with everolimus has shown promise in the treatment of sporadic TNBCs and could be equally beneficial in the treatment of ovarian cancer.^30^ Thus, we proposed a phase 1 clinical trial that aims to investigate the safety profile and effectiveness of niraparib and everolimus in patients with advanced ovarian or breast cancers.

## 2 METHODS

### 2.1 Study design and treatment

This is an open‐label, single‐center, dose escalation phase 1b cohort study to determine the feasibility and tolerability of the combination of daily niraparib and daily or thrice weekly everolimus for a 28‐day cycle in patients with advanced gynecologic malignancies and triple negative breast cancer. The primary objective was to determine safety and tolerability, maximum tolerated dose (MTD), and to recommend a phase 2 dose (RP2d) of niraparib and everolimus. Secondary objectives were to assess the toxicity of the combination of niraparib and everolimus and to determine the objective response rate (according to RECIST 1.1 response criteria) and clinical outcomes. Exploratory endpoints included the assessment of molecular aberrations, using tumor DNA and/or cell‐free DNA (cfDNA) and describing symptom occurrence/severity and Health Related Quality of Life (HRQOL) as measured by the TRSC and HRQOL‐LASA, respectively.^33^, ^34^

The trial adhered to the principles of the Declaration of Helsinki, Good Clinical Practice guidelines, and all local laws. The study protocol was approved by the local Institutional Review Board (IRB) and listed on clinicaltrials.gov (NCT03154281). All patients provided written informed consent. The trial was conducted according to a standard 3 + 3 design with four predetermined dosing levels, resulting in a maximum sample size of *n* = 24 subjects. A traditional dose escalation design was used, beginning with the lowest dose level and escalating to the maximum allowable dose level as specified in the protocol. Adverse events were defined using the Common Toxicity Criteria v. 4.03. DLTs were defined as any clinically significant AEs that could possibly be related to study treatment occurring during cycle 1 and included as follows: any death not clearly due to underlying disease or extraneous causes; AST or ALT elevations >3× the upper limit of normal (ULN) and total bilirubin elevation >2× ULN without initial findings of cholestasis (elevated serum alkaline phosphatase); Grade 3 or greater nonhematologic toxicity despite adequate treatment (with the exception of hyperglycemia, rash, nausea, and vomiting if not adequately treated with supportive measures); any grade 3 or greater hematologic toxicity that failed to resolve to at least grade 1 while the study drug was held; grade 4 neutropenia lasting >7 days without growth factor support, or associated with a fever >38.5°C, or a systemic infection; grade 3 thrombocytopenia with bleeding; grade 4 thrombocytopenia >7 days, any other grade 4 hematologic toxicity; inability to administer at least 75% of the doses of niraparib/everolimus in cycle 1 due to drug‐related toxicity; any clinically significant occurrence deemed by the investigator or sponsor that placed the patient at undue safety risk.

Patients were divided into the following 4 cohorts (each cycle was 28 days long):

*Cohort 1*: Everolimus 5 mg daily 3 days per week on Mondays, Wednesdays, and Fridays.

Niraparib 100 mg daily.

*Cohort 2*: Everolimus 5 mg daily 3 days per week on Mondays, Wednesdays, and Fridays.

Niraparib 200 mg daily.

*Cohort 3*: Everolimus 2.5 mg daily.

Niraparib 200 mg daily.

*Cohort 4*: Everolimus 5 mg daily.

Niraparib 200 mg daily.

### 2.2 Participants

Patients were eligible if they were ≥18 years old, PARP naïve, and had either a gynecologic malignancy or breast cancer (triple negative or hormone receptor positive only) refractory/intolerant to all therapies known to confer clinical benefit in the advanced or metastatic setting or if the patient's clinical team and the PI believed that the study treatment gave the patient the best chance for clinical benefit. Other criteria for all patients included as follows: having an Eastern Cooperative Oncology Group (ECOG) performance status of ≤2; adequate organ function, defined as an absolute neutrophil count ≥1500/μL, platelets ≥125,000/μL, hemoglobin ≥10 g/dL, serum creatinine ≤1.5 × ULN, or calculated creatinine clearance ≥60 mL/min using the Cockcroft‐Gault equation, total bilirubin ≤1.5 × ULN or direct bilirubin ≤1 × ULN, aspartate aminotransferase and alanine aminotransferase ≤2.5 × ULN unless liver metastases are present, in which case they must be ≤5 × ULN; female patients of childbearing potential had to have a negative serum pregnancy test (beta hCG) at screening; female patients with childbearing potential had to agree to use an acceptable method of birth control (excluding hormonal birth control methods) for 72 h prior to initiation of therapy and to continue its use during the study and for at least 30 days after the final dose or as directed by the Investigator Brochure, and male patients had to agree to use an acceptable form of birth control from study Day 1 through at least 90 days after the final dose or as directed by the Investigator Brochure.

Inclusion criteria specific to cancer type also were in place. Patients with breast cancer needed to have measurable disease per RECIST 1.1. criteria. Patients with ovarian cancer must have had appropriate surgical management for their disease and needed to be platinum resistant (recurrence within 6 months of the last platinum‐containing regimen) or be refractory to platinum‐containing regimens. Patients with endometrial, cervical, or any other advanced gynecologic malignancy must have already received or not been a candidate for all therapy proven to have a survival benefit.

Exclusion criteria included as follows: Her2+ breast cancer measured by standard IHC or FISH testing, simultaneous enrollment in any other interventional clinical trial, major surgery ≤3 weeks of starting the study, investigation therapy administered ≤4 weeks, or within a time interval less than at least 5 half‐lives of the investigational agent, whichever is longer, prior to the first scheduled day of dosing in this study; previous radiotherapy encompassing >20% of the bone marrow; prior treatment with a known PARP inhibitor or participation in a study where any treatment arm included administration of a known PARP inhibitor; known hypersensitivity to the components of niraparib, everolimus, rapamycin analogues, or the excipients; immunocompromised status (patients with splenectomy were allowed); patients with any known, persistent > Grade 2 toxicity from prior cancer therapy; known, persistent (>4 weeks), ≥Grade 3 hematological toxicity or fatigue from prior cancer therapy; (a history of a transfusion platelets or red blood cells) ≤4 weeks of the first dose of study treatment; current evidence of any condition, therapy, or laboratory abnormality (including active or uncontrolled myelosuppression [i.e., anemia, leukopenia, neutropenia, and thrombocytopenia]) that might confound the results of the study or interfere with the patient's participation for the full duration of the study treatment or that makes it not in the best interest of the patient to participate; cancer ≤2 years prior to randomization (except basal or squamous cell carcinoma of the skin that has been definitively treated); known, symptomatic brain or leptomeningeal metastases; patients considered a poor medical risk due to a serious, uncontrolled medical disorder, nonmalignant systemic disease, or active, uncontrolled infection; a psychiatric disorder that prohibits obtaining informed consent; a known history of myelodysplastic syndrome (MDS) or acute myeloid leukemia (AML); a female with positive serum pregnancy test ≤3 days prior to study drug administration, who is breastfeeding, or is planning to conceive children within the projected duration of the study treatment; uncontrolled or poorly controlled hypertension; taking ACE inhibitors; taking strong or moderate CYP3A4/PgP inhibitors or strong CYP3A4/PgP inducers.

### 2.3 Assessments

Screening tests were performed within 28 days prior to study registration and included the following: demography and medical history including documentation of prior regimen; vital signs including temperature, blood pressure, pulse, respiration rate, and oxygen saturation; concomitant medications; ECOG performance status, laboratory assessment (complete blood count with differential and platelet count [CBCD], lipid panel, ALT/SGPT, AST/SGOT, alkaline phosphatase, total bilirubin, and creatinine clearance [calculated using the Cockcroft formula] or creatinine and albumin; serum pregnancy test if indicated, tumor assessment according to RECIST 1.1, and collection of whole blood samples for cytogenetic analysis if required).

The following evaluations were performed during active therapy: physical exam (including vital signs), ECOG performance status, concomitant medications, laboratory analyses (CBCD, comprehensive metabolic panel), blood pressure monitoring; Adverse Event Monitoring; completion of questionnaires (TRSC and HRQOL‐LASA). CBCD was performed weekly from D14 until the end of the first cycle (D28) and then monthly thereafter. Blood pressure monitoring continued weekly for 2 cycles (8 weeks) through Cycle 3 Day 1. After the completion of 2 cycles (56 ± 7 days), patients were assessed for disease response utilizing the same analysis that was used at baseline and then again at 16 weeks (112 ± 7 days) if the patient had stable disease or better. If the patient continued on trial, scans were performed every 8–12 weeks thereafter at the discretion of the investigators.

End of treatment assessments (within 7 days of last dose) included a lipid panel, whole blood sample for cytogenetic analysis, Adverse Event Monitoring, and completion of questionnaires (TRSC and HRQOL‐LASA). Ongoing assessments included as follows: a disease status assessment performed every 8–12 weeks (i.e., every 56–94 ± 7 days) at the discretion of the investigators, adverse events monitored for 30 days following the last dose, and survival status monitored every 8 weeks for at least 2 years following the last dose.

Radiographic response was evaluated according to RECIST 1.1 criteria in all patients with measurable disease. Tumor assessments for evaluation of response were conducted every 8 weeks × 2, and then every 8–12 weeks thereafter until study discontinuation or disease progression, whichever was later. All sites of disease were evaluated using the baseline assessment methods. Confirmatory assessment of complete response (CR) or partial response (PR) was performed no <4 weeks after the initial documentation of response.

Tumor marker assessments for those patients without measurable disease (GYN only) that had elevated levels on the last prior regimen were taken at baseline and approximately every 8 ± 2 weeks along with tumor assessments during the study treatment period. All tumor marker assessments were performed by the same laboratory for each patient. All target lesions were measured by consistent imaging techniques throughout the study. The same technique was used for each evaluation in an individual patient. Results were recorded in the CRF. Copies of the scans were available for review. All patients who received at least two treatment cycles and underwent at least one on‐study disease assessment or experienced early progression were considered evaluable for response.

### 2.4 Biomarker analysis

To assess whether certain molecular aberrations responded more favorably to the combination, tissue from a new or existing biopsy was sent for somatic sequencing in all patients. Additionally, sequencing DNA from some patients' blood samples using cfDNA were used as part of the analysis. In all cases, this analysis was carried out in a retrospective manner and patients were not selected based on their molecular aberrations for this study.

### 2.5 Statistical analysis

The total sample size was dependent on the number of patients treated in each dosing level (DL) and the DLTs observed. To be evaluable for safety, PFS, and OS, patients should have received at least one dose of niraparib and everolimus. To be evaluable for antitumor activity, patients should have completed at least one cycle of treatment and undergone at least one postbaseline tumor measurement.

Descriptive statistics were used for demographic, safety, and efficacy. Median PFS was determined by the Kaplan–Meier method. Differences between median survivals were tested with the log‐rank test. Response rate and relationship with genomic alterations were assessed using a two‐sided Fisher Exact test. In all statistical tests, an effect was considered as statistically significant if the *p*‐value of its corresponding statistical test was ≤5%.

## 3 RESULTS

### 3.1 Patient demographics

Between 2017 and 2021, 14 patients were enrolled. Primary tumor types were high‐grade serous ovarian cancer (*n* = 12), clear cell ovarian cancer (*n* = 1) and triple negative breast cancer (*n* = 1). Median age was 65 years (range 51–77). The main patient characteristics are summarized in Table 1. The average number of prior lines of therapy were 4 (range 2–10). All patients with ovarian cancer were platinum resistant/refractory except for 2 patients who were medically incapable of receiving platinum due to severe hypersensitivity despite prior attempts at desensitization and switching to other platinum compounds. Platinum sensitivity/resistance did not apply to the one enrolled breast cancer patient.

**TABLE 1:** Patient characteristics.

Study ID	Age	Cohort	Lines of Therapy	Cancer Type	Stage	Select genomic aberrations				ECOG/Performance Status
						*BRCA1/2* (germline)	HRD status	BRCA1/2 pathway (somatic)	PI3K/AKT/mTOR pathway	
1	66	1	4	HGSOC	IV	Not tested	Not tested	N	PIK3CA K111E	1
2^c^	51	1	7^a^	HGSOC	III	Not tested	Not tested	N	PTEN loss	1
3	71	1	6	HGSOC	II	Not tested	Not tested	N	PIK3CA E545K	1
4	69	1	2	HGSOC	IV	Negative	Not tested	BRIP1 exon 3 del	N	0
5^c^	70	2	6	HGSOC	III	Negative	Not tested	N	N	0
6	77	2	1	HGSOC	IV	Negative	Not tested	N	N	0
7	61	2	6^a^	BC	IV	negative	not tested	BRCA2 R278H	PIK3CA E545K	0
8	63	2	4	HGSOC	III	Not tested	Not tested	N	PIK3CA R88Q	0
9	51	2	0	CCOV	I	Not tested	Not tested	N	PIK3CA N345K	1
10^b^	57	2	2	HGSOC	IV	Negative	Not tested	N	N	1
11	62	1	3	HGSOC	III	Negative	Positive (FoundationONE CDx HRD)	N	N	1
12	69	1	2	HGSOC	IV	Negative	Negative (TumorNext‐HRD with OvaNext)	N	N	1
13	71	1	3	HGSOC	III	Negative	Inconclusive (TumorNext‐HRD)	BRCA1 loss	N	1
14	57	1	3	HGSOC	IV	Negative	Not tested	N	N	0

Abbreviations: BC, breast carcinoma; CCOV, clear cell ovarian cancer; HGSOC, high‐grade serous ovarian cancer.

^a^ Patient 2 and Patient 7 had everolimus prior to enrollment.

^b^ Patient 10 sequenced 3 and 9 months post initiation of therapy.

^c^ Patients 2 and 5 were intolerant of platinum agents.

### 3.2 Treatment

The median number of cycles administered was 4 and the total number of cycles was 52. Reasons for discontinuation of treatment were PD (*n* = 11), and toxicity (*n* = 3). Among the 14 patients enrolled, all were evaluable for DLT. Two DLTs were experienced in cohort 2 (niraparib 200 mg daily and Everolimus 5 mg 3 days per week) with 1 patient experiencing thrombocytopenia and one patient experiencing severe hypertension. After these 2 DLTs were noted, we de‐escalated back down to the cohort 1 dose and schedule and 4 additional patients were enrolled to confirm the MTD. A summary of the DLTs is described in Table 2.

**TABLE 2:** DLTs by DL.

Cohort	Everolimus dose	Niraparib dose	Patient (*n*)	DLTs (*n*)	Type of DLT
1	5 mg daily on M, W, F	100 mg daily	8	0	
2	5 mg daily on M, W, F	200 mg daily	6	2	Patient 5: Thrombocytopenia Patient 10: Hypertension

### 3.3 Safety

All patients were evaluable for safety and the complete list of TEAEs of any grade are summarized in Table 3. 155 total TEAEs were reported and noted to occur in all patients. The most common laboratory‐ related TEAEs were anemia (11.6%), thrombocytopenia (9.7%), leukopenia (8.4%), elevated alkaline phosphatase (5.8%), neutropenia (5.2%) and elevated alanine aminotransferase (3.9%). The most common nonlaboratory TEAEs of any grade were fatigue (7.8%), hypertension (7.1%), nausea (4.6%) and mucositis (3.9%). 17 TEAEs were grade 3 or grade 4. The most frequent grade 3 or greater TEAEs were thrombocytopenia (2.6%), hypertension (2.6%), anemia (2%), fatigue (1.3%), neutropenia (0.6%), and elevated alkaline phosphatase (0.6%). There were no treatment‐related deaths.

**TABLE 3:** AE s by cohort.

	Cohort 1^a^		Cohort 2			Totals			Total	Percent
	Gr. 1&2	Gr. 3	Gr. 1&2	Gr. 3	Gr. 4	Gr. 1&2	Gr. 3	Gr. 4	All Grades	All Grades
Anemia	7	1	8	2	—	15	3	—	18	11.61%
Platelet count decreased	4	—	7	3	1	11	3	1	15	9.68%
White blood cell decreased	2	—	11	—	—	13	—	—	13	8.39%
Fatigue	3	2	7	—	—	10	2	—	12	7.74%
Hypertension	4	2	3	2	—	7	4	—	11	7.10%
Alkaline phosphatase increased	6	1	2	—	—	8	1	—	9	5.81%
Neutrophil count decreased	—	—	6	1	1	6	1	1	8	5.16%
Nausea	3	—	4	—	—	7	—	—	7	4.52%
Alanine aminotransferase increased	5	—	1	—	—	6	—	—	6	3.87%
Mucositis oral	3	—	3	—	—	6	—	—	6	3.87%
Headache	2	—	3	—	—	5	—	—	5	3.23%
Anorexia	1	—	3	—	—	4	—	—	4	2.58%
Constipation	1	—	3	—	—	4	—	—	4	2.58%
Rash maculo‐papular	2	—	2	—	—	4	—	—	4	2.58%
Hypokalemia	3	—	—	—	—	3	—	—	3	1.94%
Arthralgia	—	—	2	—	—	2	—	—	2	1.29%
Aspartate aminotransferase increased	2	—	—	—	—	2	—	—	2	1.29%
Creatinine increased	1	—	1	—	—	2	—	—	2	1.29%
Diarrhea	2	—	—	—	—	2	—	—	2	1.29%
Dry mouth	1	—	1	—	—	2	—	—	2	1.29%
Hyponatremia	2	—	—	—	—	2	—	—	2	1.29%
Insomnia	—	—	2	—	—	2	—	—	2	1.29%
Muscle weakness lower limb	2	—	—	—	—	2	—	—	2	1.29%
Myalgia	2	—	—	—	—	2	—	—	2	1.29%
Acidosis	—	—	1	—	—	1	—	—	1	0.65%
Bruising	—	—	1	—	—	1	—	—	1	0.65%
Dysgeusia	—	—	1	—	—	1	—	—	1	0.65%
Epistaxis	—	—	1	—	—	1	—	—	1	0.65%
Hypocalcemia	1	—	—	—	—	1	—	—	1	0.65%
Hypophosphatemia	—	—	—	1	—	—	1	—	1	0.65%
Nail ridging	—	—	1	—	—	1	—	—	1	0.65%
Oral dysesthesia	—	—	1	—	—	1	—	—	1	0.65%
Sinus tachycardia	—	—	1	—	—	1	—	—	1	0.65%
Sore throat	—	—	1	—	—	1	—	—	1	0.65%
Urinary tract infection	—	—	1	—	—	1	—	—	1	0.65%
Vomiting	1	—	—	—	—	1	—	—	1	0.65%
				Totals		138	15	2	155	

^a^ No grade 4 adverse events noted.

### 3.4 Efficacy

**FIGURE 1:**
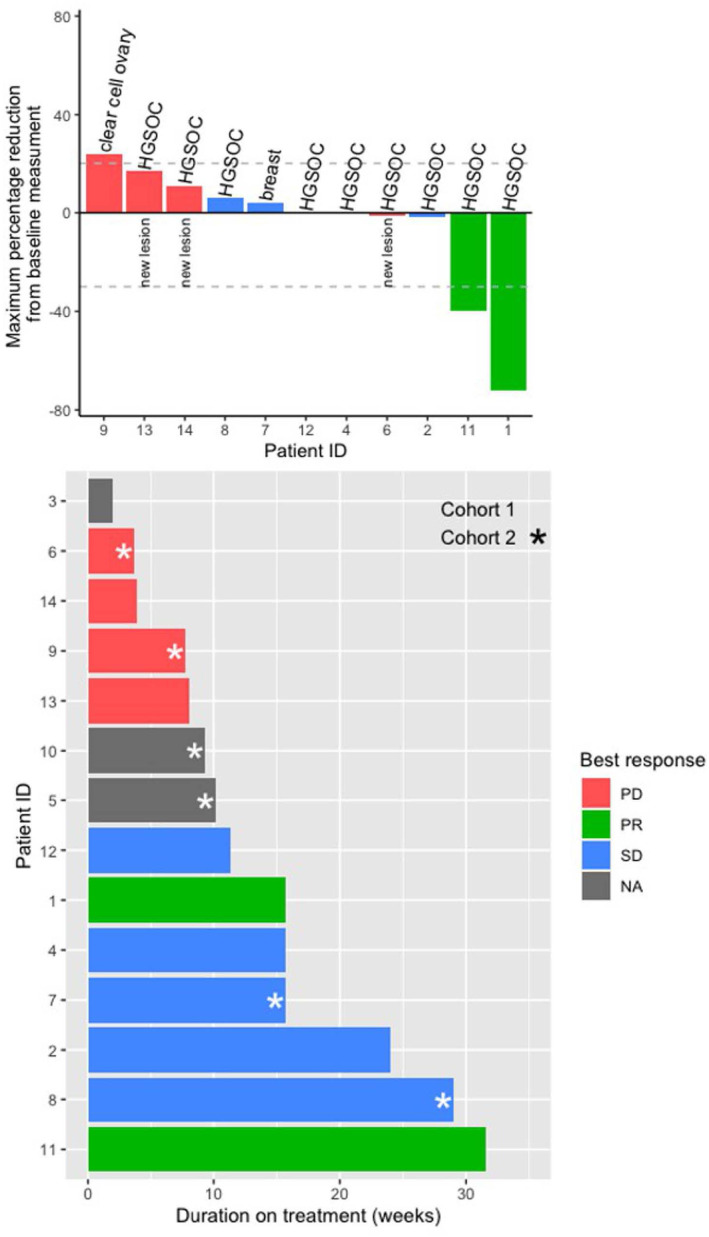
Treatment outcomes.

11 out of 14 patients were evaluable for efficacy. The ORR was 18% and the CBR was 45% (11/14 patients: 2 PR, 3 SD). There were no complete responses and 6 patients had PD after completion of two cycles of treatment. Of the patients with SD or better, most experienced moderate benefit at the doses utilized. At data cutoff median PFS was 6 months and median OS is approximately 18 months with 3 patients still alive at the time of composition. Response to treatment and duration of the response are summarized in Figure 1 and Table 4, respectively.

**TABLE 4:** Best response.

*N* (%)	All patients (*n* = 14)
Partial response	2 (14%)
Stable disease	5 (36%)
Progressive disease	5 (36%)
Not evaluable	2 (14%)

### 3.5 Biomarker

**FIGURE 2:**
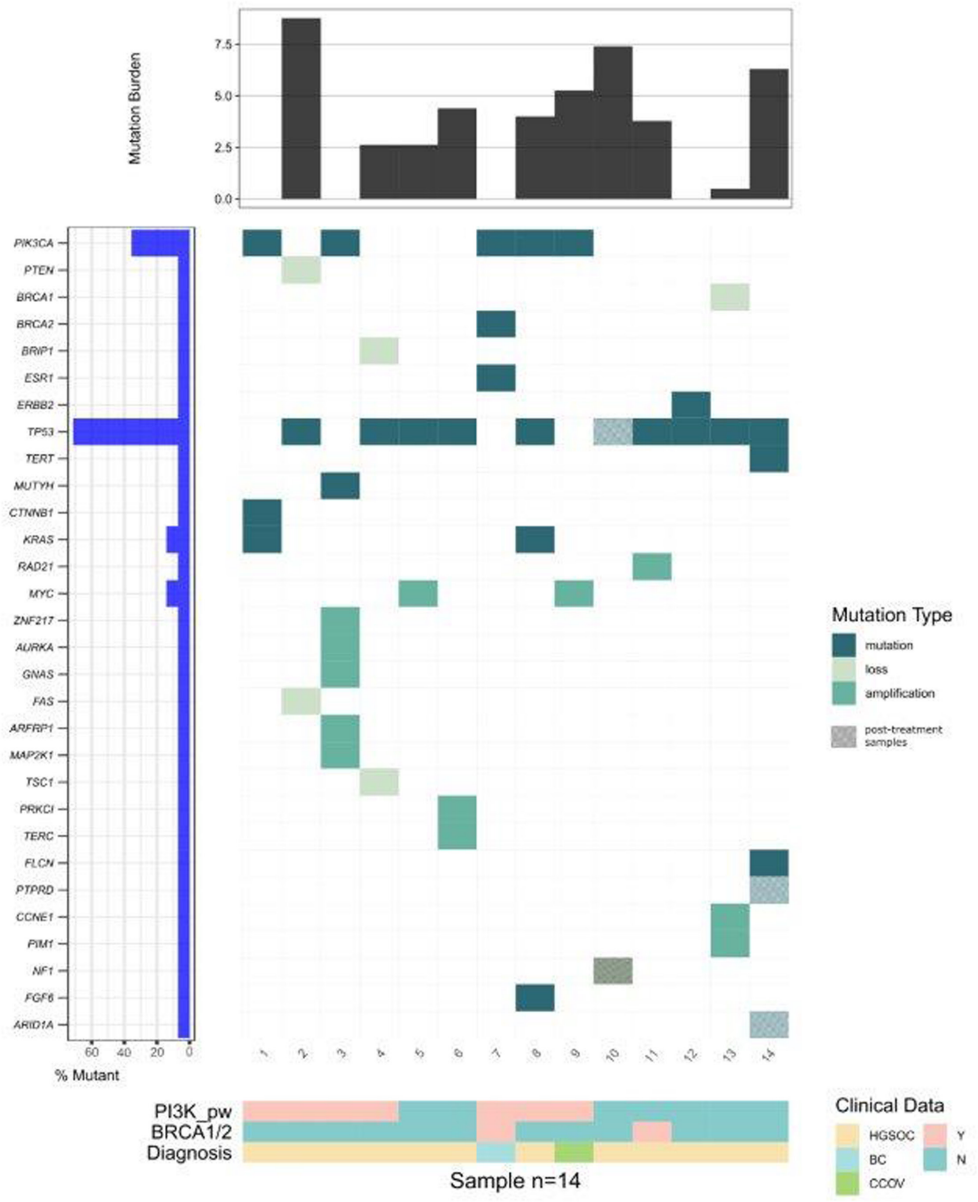
Molecular analysis.

Including only the patients ultimately tested for germline BRCA1/2 aberrations (*n* = 9), no patient had a known germline mutation detected (Table 1). 6/14 patients had a mutation in the PI3K/AKT/mTOR pathway with one patient having a loss of PTEN, and five patients had a PIK3CA mutation with one of the patients having a coexisting BRCA2 mutation. Three patients were found to have mutations in BRCA1/2 and BRIP1, respectively. Another patient tested positive for HRD, but did not have any mutations in either the BRCA1/2 or the PI3K/AKT/mTOR pathway. The remaining five patients had no mutations in either pathway and were not positive for HRD (one patient) or not tested (four patients). The overall response rate for patients with positive results for PI3K, BRCA1/2 pathway aberrations, or HRD was 75% (*n* = 6), while it was 25% (*n* = 2) for negative patients. No clear correlation linking response to any molecular aberration was found in this study. Refer to Figure 2 for a summary of the molecular data.

Sequencing of a post‐treatment tumor sample from patient 14 showed a de‐novo PTPRD c.3715‐1G > T splice region variant.

### 3.6 Patient‐reported outcomes

All patients completed the questionnaires at baseline and the compliance rate was over 95% throughout the trial. The PRO data will be published and presented separately.

## 4 DISCUSSION

Therapeutic options for heavily pretreated platinum resistant ovarian cancer patients currently remains limited. Single agent PARP inhibitor activity is poor in BRCA wildtype patients with olaparib demonstrating a 13% response rate in the Phase 2 CLIO study^35^ and niraparib an 8% response rate in the Phase 2 QUADRA study.^36^ Combination therapy also appears challenging, even in the less heavily pretreatment platinum‐sensitive population. The recently completed NRG‐GY004 study evaluating single agent olaparib versus olaparib/cederanib versus standard chemotherapy failed to demonstrate an improvement in PFS over chemotherapy, but did demonstrate a significant decrease in the patient‐reported outcomes (PROs) score in the BRCA wildtype cohort.^37^ While clinical activity with the olaparib/cederanib arm was demonstrated in the BRCA mutant cohort, in the current treatment environment, BRCA mutant patients will most likely have received PARP maintenance as part of their upfront therapy, and retreatment with PARP inhibitors is currently cautioned due to accumulating data of increased risk for acute myeloid leukemia and myelodysplastic syndrome. For this challenging patient population, perhaps newer options for treatment such as Antibody Drug Conjugates (ADCs) may offer a glimmer of hope. In the recently completed Phase 3 SORAYA trial the ADC mirvetuximab soravtansine‐gynx demonstrated an ORR of 32.4% in folate receptor alpha high patients and a median OS of 15 months.^38^ Further treatment options for this patient population are sorely needed.

Based upon preclinical studies showing additive or synergistic effects of combining PARP inhibitors with a number of chemotherapeutic agents, multiple clinical trials assessing PARP inhibitor combination regimens have been conducted.^5^, ^7^, ^12^, ^15^, ^23^, ^24^, ^25^, ^30^, ^31^, ^32^, ^39^, ^40^ However, most combinations have generally shown exacerbation of toxicity suggesting although there may be chemopotentiation there is not a wide therapeutic index. Matulonis and colleagues initially reported the combination between BKM120, a nonselective PI3K inhibitor, and olaparib in patients with breast and ovarian cancers was possible with dose attenuation of the BKM120 in 2017 as we were initiating this trial.^40^

We conducted a phase 1 dose escalation investigation to assess the safety, tolerability, and preliminary antitumor activity of the combination of niraparib and everolimus in patients with advanced breast and gynecological malignancies. All participating patients were heavily pretreated with an average of four regimens prior to enrollment and were considered to have platinum resistant disease. Two patients (14%) had also previously received everolimus, but no patient had received prior therapy with a PARP inhibitor. All patients had comprehensive molecular profiling performed prior to enrollment, but study selection was not based predominantly upon an assessment of biomarker status. The combination did not elucidate any new or unexpected adverse events relative to the expected side effects from either compound alone, but the severity, rate of onset, and magnitude of the hypertension was noteworthy. One patient experienced prolonged thrombocytopenia as a DLT in cohort 1, but this was considered a likely adverse event in a heavily pretreated population receiving a PARP inhibitor and everolimus. An additional patient experienced rapid onset hypertension as a DLT in cohort 2 and per protocol, another 4 patients were enrolled at the lowest dose. Severe hypertension was again experienced by another patient and the study was subsequently halted. While hypertension is a known side effect of everolimus (17–30% per package insert^41^), severe hypertension is expected to be less than 1%. Hypertension is also reported to be a side effect of niraparib (18%–21% per package insert^42^) with hypertensive crisis reported postmarketing at <1%. Based on the fact that two patients (14%) experienced severe hypertension, we conclude severe hypertension is a probable side effect of this combination, even at low doses. The most frequent TEAEs of grade 3 or greater were hypertension (36%), anemia (17%), and fatigue (17%).

The combination demonstrated modest efficacy with observed antitumor activity with the doses utilized. The ORR was 18% and the CBR was 45%. PFS was 6 months, but interestingly the OS is currently at 18 months as 3 patients remain alive with disease at the time of data cutoff. We were not able to reach a dose and schedule of the combination that included a daily dose of everolimus due to the development of the hypertension. The expected occurrence of cytopenia proved to be similar to what we have observed clinically with single agent PARP inhibitors.

The strength of this study was the successful conduct of a Phase I dose escalation study in a heavily pretreated population of patients with compounds not previously investigated in combination. All patients were naïve to PARP inhibitors and only 2 patients had prior exposure to PI3K alpha blockers. Comprehensive molecular profiling was performed on all patients enrolled, but no clear conclusions could be made linking outcome with any biomarker due to the limited sample size. The limitations of this study were its early conclusion based on the toxicity of this combination of drugs. This limited the ability to interpret the efficacy of PARP and PI3K alpha blockade in advanced breast and ovarian cancer. Perhaps at high doses greater efficacy might have been realized. Finally, very few breast patients were enrolled in this study limiting assessment of this drug combination in the breast population.

## 5 CONCLUSIONS

In conclusion, the combination of everolimus and niraparib even at the attenuated doses studied, proved too toxic to continue beyond cohort 1. This suggests the combination of drugs would not be feasible to evaluate in further studies given these dosing limitations. This limitation possibly blunted the efficacy of the combination as PFS was modest, but OS was surprisingly robust due to three patients with ovarian cancer remaining alive with platinum refractory disease. Exploration and potential leveraging of dual blockade of the PARP and PI3K/AKT/mTOR pathway awaits different combinations and treatment schedules. Dual blockade of the PI3K pathway would seem to be essential as has been demonstrated in a prior publication by our group evaluating dual PI3K and mTOR inhibition.^40^, ^41^ In our prior study, in heavily pretreated endometrial, ovarian, and breast cancer patients, dual blockade resulted in 3 CR, 4 PR and 4 SD. Without at least dual blockade of the PI3K pathway it would seem the response rate is limited and short lived. The Phase III EPIK‐O trial evaluating the PI3K alpha blocker alpelisib and olaparib in high‐grade serous ovarian cancer patients without germline BRCA mutations has recently completed enrollment and it will be intriguing to see if this trial has more success with single agent blockade of the PI3K/AKT/mTOR pathway. With the publication of EPIK‐O, perhaps further clarity on the role of combination PI3K blockade with PARP inhibition will be forthcoming.

## AUTHOR CONTRIBUTIONS

**David Starks:** Formal analysis (equal); investigation (equal); supervision (equal); writing – original draft (equal); writing – review and editing (equal). **Luis Rojas‐Espaillat:** Investigation (equal); writing – review and editing (equal). **Tobias Meissner:** Formal analysis (equal); writing – original draft (equal); writing – review and editing (equal). **Rachel Elsey:** Formal analysis (equal); writing – original draft (equal); writing – review and editing (equal). **Bing Xu:** Formal analysis (equal); writing – original draft (equal); writing – review and editing (equal). **Maria Koenen:** Writing – original draft (equal). **Shelley Feng:** Writing – original draft (equal). **Annika VanOosbree:** Writing – original draft (equal). **John Slunecka:** Writing – original draft (equal). **John Lee:** Writing – original draft (equal); writing – review and editing (equal). **Casey Williams:** Conceptualization (lead); data curation (equal); formal analysis (equal); funding acquisition (lead); investigation (lead); methodology (lead); project administration (lead); supervision (lead); writing – original draft (equal).

## Data Availability

All relevant data generated as part of this study are being presented in this manuscript.
